# Cleft Care Companion: An innovative app to educate and connect patients with a cleft and their families to treatment centres

**DOI:** 10.7189/jogh.13.03048

**Published:** 2023-09-01

**Authors:** Abel M Smerica, Ewa Rumprecht, Grace Peters, Felicity V Mehendale

**Affiliations:** 1 Division of Plastic Surgery, Michael E. DeBakey Department of Surgery, Baylor College of Medicine, Houston, Texas, USA; 2 Division of Plastic Surgery, Department of Surgery, Texas Children’s Hospital, Houston, Texas, USA; 3 Smile Train, New York City, New York, USA; 4 Global Cleft Lip and Palate Research Programme, Centre for Global Health, Usher Institute, University of Edinburgh, Edinburgh, Scotland, UK

Despite the availability of experienced local clinicians across low- and middle-income countries (LMICs), many patients and families with cleft lip and/or palate (CLP) remain unaware of the existence of comprehensive cleft care (CCC) and its access. Furthermore, limited awareness even among health care workers, leads to preventable morbidity, delays in receiving treatment, and the perception that the sole option is to access cleft care from visiting surgical missions [1]. Traditionally, the approach to identifying a child with a CLP in resource-limited settings involves community outreach, social workers, or health care providers alongside brochures, advertising, or word-of-mouth referrals. With the availability of technology and global internet accessibility increasing tremendously within the last years, technology may be leveraged to reach further and remoter regions than before. According to the latest connectivity reports, 95% of the global population lives within the footprint of at least a 3G mobile internet connection, with just over four billion people (an estimated 55%) already using mobile internet [2]. The affordability of internet-capable devices has also been generally increasing in the last six years, allowing for more connectivity, even in the LMIC setting [2]. To Smile Train, the world’s largest cleft organisation, a digitally connected world means that further opportunity exists to provide CLP treatment and care through education, outreach, and patient referral. Cleft Care Companion, released in mid-2021, is a mobile app designed to be a combined electronic brochure, educational tool, and referral tool for use by anyone with an internet-connected Android [3] or iOS [4] device. Originally designed to take the form of an electronic brochure, the novel Cleft Care Companion mobile application has been created to take cleft care to the furthest corner of the world.

## CLEFT CARE COMPANION HISTORY

Smile Train funds and supports local cleft teams to deliver CCC to patients in LMICs, where access to surgery and medical resources may be highly limited. Smile Train’s partner network of more than 1100 treatment centres in more than 70 countries has treated over 1.5 million cleft patients in LMICs free of charge [5,6]. Early referral of babies with clefts to specialised CCC centres as close as possible to their homes is essential to avoid preventable morbidity and mortality and to ensure optimal treatment outcomes [7]. While historical approaches of brochures, radio broadcasts, and clinician referrals have led to an increase in referrals and a decrease in age at referral, referral delays remain a concern, particularly in underserved or remote areas [8]. In an effort to expand cleft awareness and education and improve equity of access to local Smile Train-affiliated centres providing free CCC, Smile Train leveraged technology to educate and link patients to treatment centres. The mobile application, Cleft Care Companion, was envisioned with the target audience of global non-governmental organisations (NGOs), health care workers, and government/public information teams working “in the field” in LMICs to be able to refer and connect patients to treatment centres.

**Figure:**
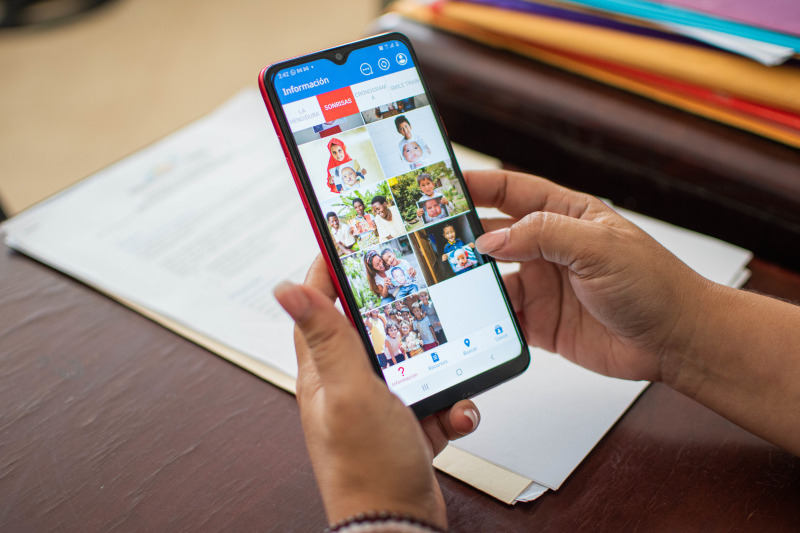
Photo: The Cleft Care Companion mobile app, available in multiple languages, demonstrated in use. Source: Courtesy of Smile Train. No author.

## IMPORTANCE OF COMMUNITY/SOCIAL WORKERS

In LMICs, the role of community health workers and social workers is essential to increasing access to basic and specialised health care [9,10]. These workers are most often the principal health care contact in remote regions and carry a trusting relationship with the regions they serve. The expansion of technology and internet connectivity allows community and social workers to use mobile devices to assist with evaluations, communication, referrals, and even data collection, having been proven as an effective way to assist with job functions [11,12]. For remote regions not covered by a larger hospital system, community health workers and social workers provide a crucial role in education, spreading awareness, and documenting the need for health care interventions. Mobile health apps have the potential to minimise and address inequalities in access to health care that are often experienced by families in remote or underserved areas.

## CLEFT CARE COMPANION APP

Cleft Care Companion is a freely available app released in mid-2021 to be used as a tool to educate patients, their families, and health care workers while enabling rapid and effective patient referral to their local CCC centres for free treatment. Its target audience includes members of the health care community, social workers, community workers, government health care officials, and patients and their families. Cleft Care Companion is an up-to-date education tool providing essential information about clefts in lay language and is available in English, Spanish, and French, with more languages to come. The app is built in 4 main sections: Information, Resources, Find, and Cases.

**Figure 1:**
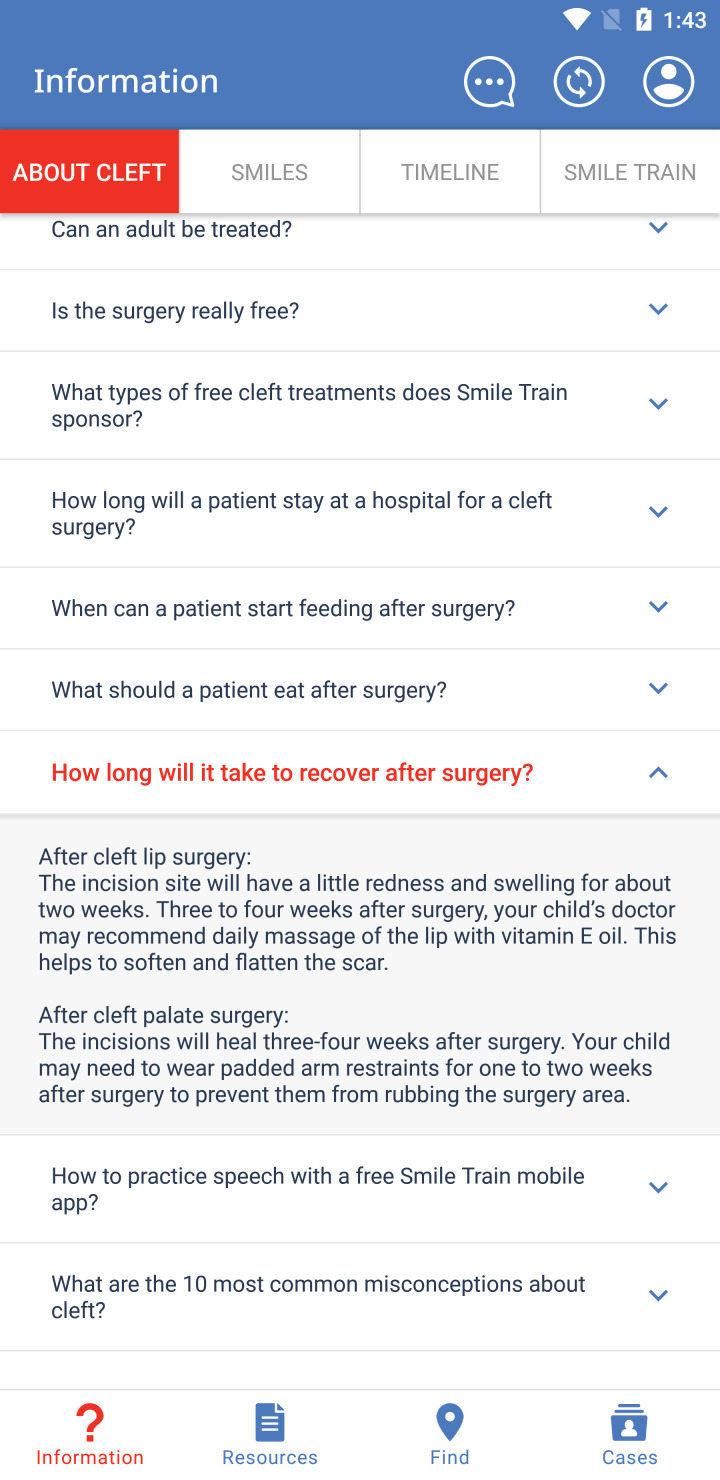
Cleft Care Companion frequently asked questions.

**Figure 2:**
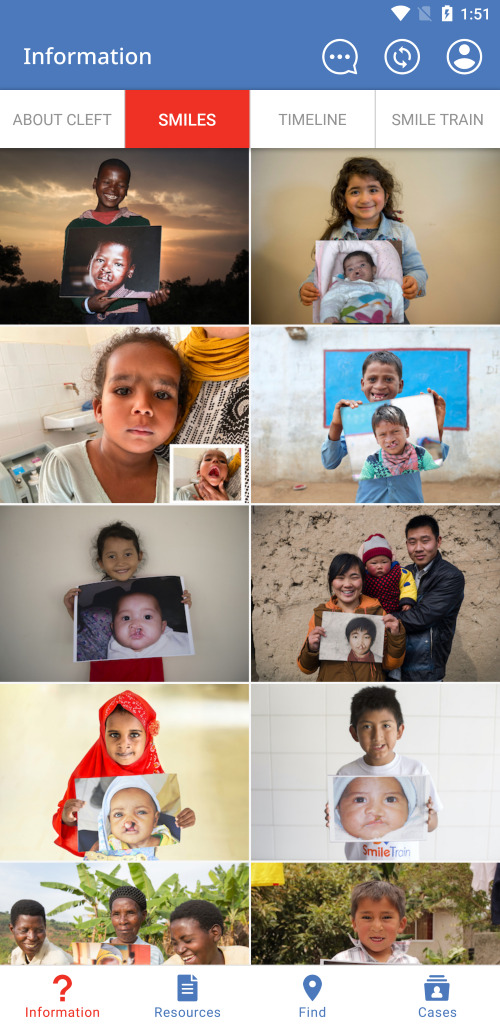
Cleft Care Companion patients.

**Figure 3:**
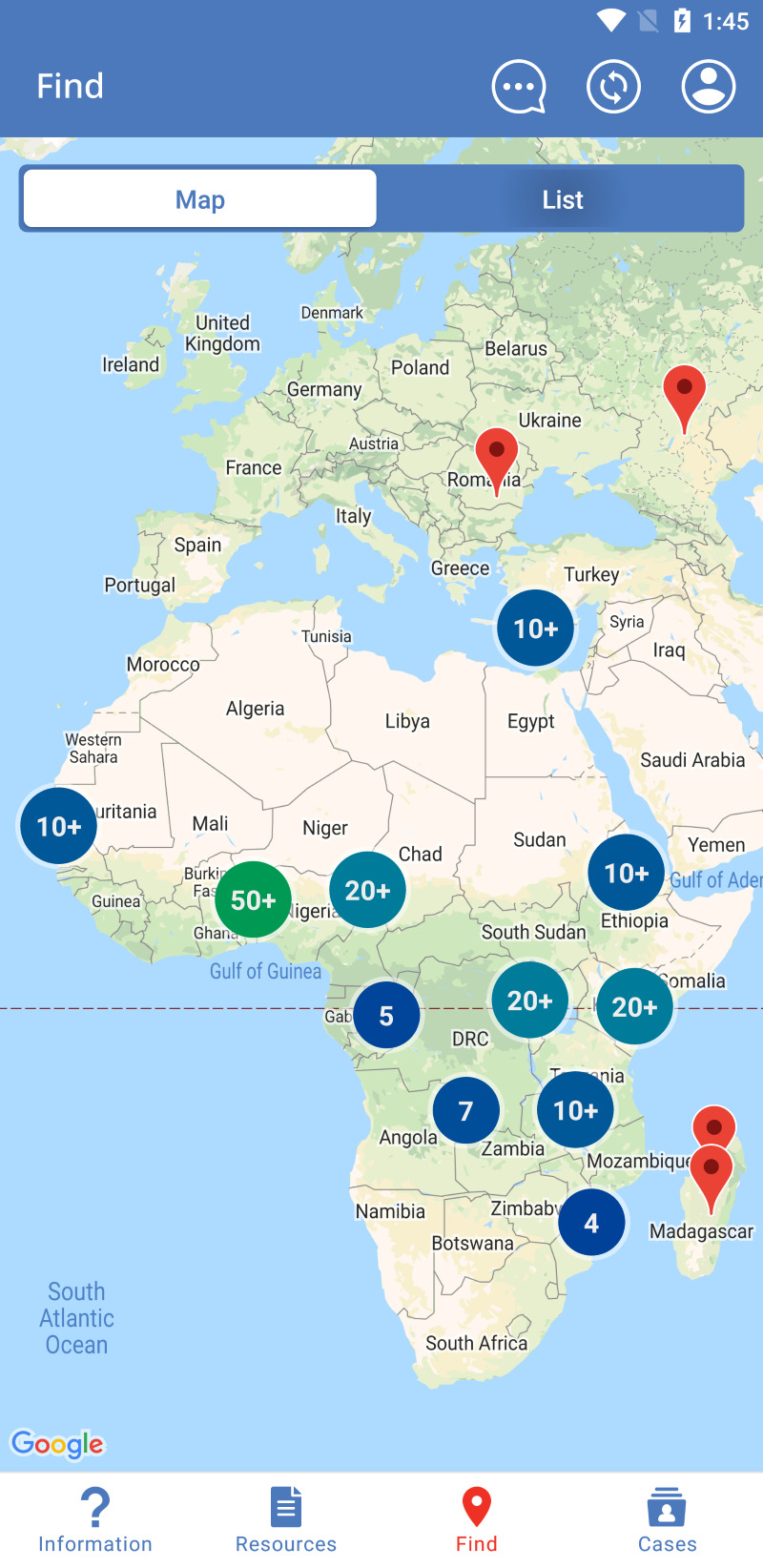
Cleft Care Companion map view and location tool to identify a treatment centre.

**Figure 4:**
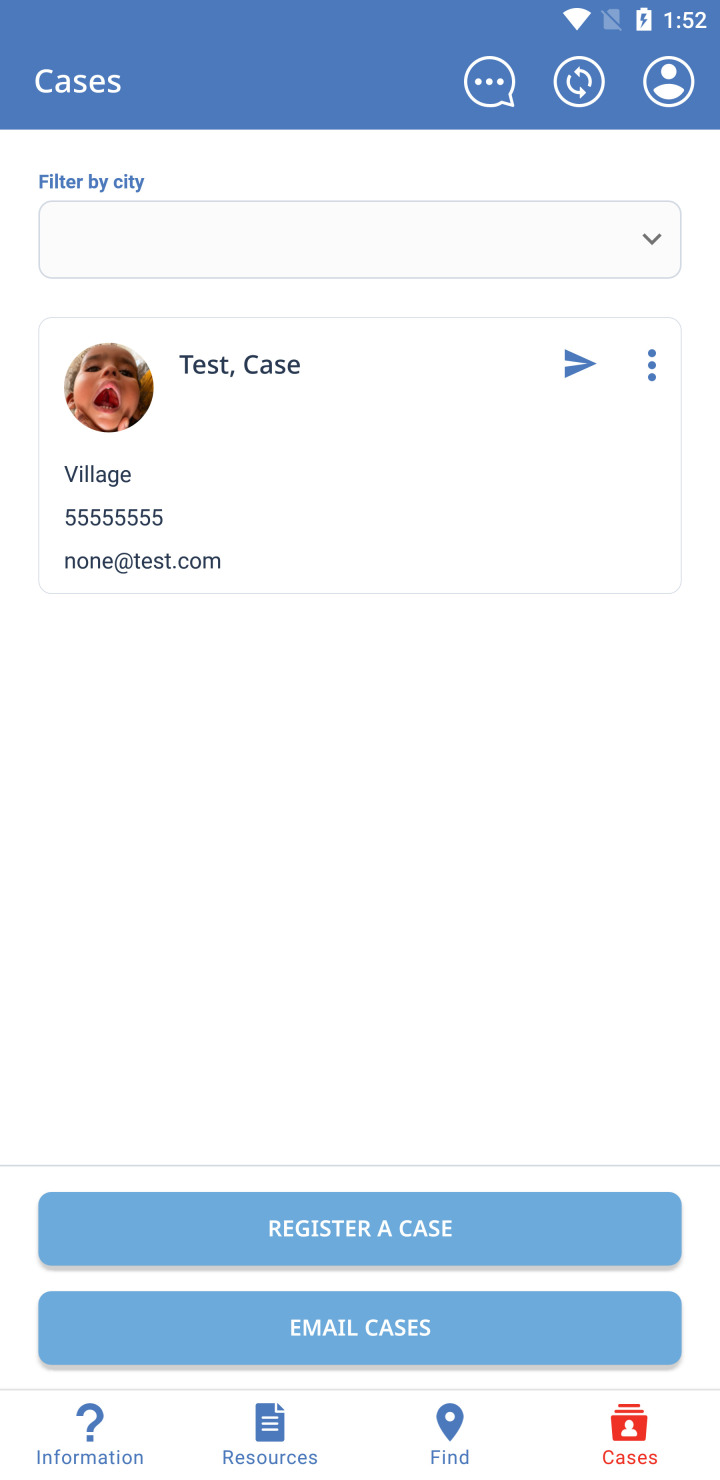
Cleft Care Companion case registration for health care and community workers.

Application usage depends on the user, with the majority of users using the Information, Resources, and Find sections. The Information section answers frequently asked questions about clefts (**Figure 1**), displays treatment timelines and milestones for patients with clefts, shows actual patients (**Figure 2**), and provides information about Smile Train. A large portion of this information can be downloaded locally for offline display or sharing in the Resources section. In the Find section, Smile Train partners are listed on a world map (**Figure 3**), utilising location services to match a treatment centre closest to the user. CCC centres have their contact info and services offered displayed to help connect patients to their nearest centre. The Cases section is designed for health care, social, or community health workers to register cases within the Cleft Care Companion system. When a health care worker or an individual in the community encounters a person with a cleft needing help, they can register the case into the app by adding personal details, location, and a picture (**Figure 4**). Registered cases are contacted by Smile Train staff to provide assistance in receiving timely treatment from the nearest centre.

There are more than 320 unique users of Cleft Care Companion originating from the app’s pilot phase. Since then, a total of 522 patient cases have been registered within Cleft Care Companion with an average of 208.8 cases registered per year, with the majority of cases being registered in the Sub-Saharan Africa region. Following upcoming awareness campaigns with community health workers and other health care professionals, these app usage metrics are expected to show an increase in app use. App metric data collection subsequently informs strategies for the distribution of the app as well as evaluating the impact of its use.

## CONCLUSION

Improving equity of access to care involves not only the ongoing building of local clinical capacity, but also ensuring that patients, the public, and health care workers are aware of how and where to access such care. The Cleft Care Companion app was developed to meet these needs, being co-designed with input from health care and Smile Train staff representing 30 countries utilising their experiences of the challenges patients face in accessing care and their recognition of the need for accessible education. Continuing work includes application use analytics and translating the app further to expand the reach of CCC services and reduce the burden of clefts worldwide.
